# BAI1: from cancer to neurological disease

**DOI:** 10.18632/oncotarget.8193

**Published:** 2016-03-19

**Authors:** Dan Zhu, Erwin G. Van Meir

**Affiliations:** Laboratory of Molecular Neuro-Oncology, Departments of Neurosurgery and Hematology and Medical Oncology, School of Medicine and Winship Cancer Institute, Emory University, Atlanta, GA, USA

**Keywords:** BAI1, tumor suppressor, synaptic plasticity

Brain-specific Angiogenesis Inhibitor 1 (BAI1) is a member of the adhesion G protein-coupled receptor (GPCR) sub-family and is mainly expressed in the brain. Structurally, BAI1 has a large N-terminus containing five thrombospondin type 1 repeats (TSR), a putative hormone-binding domain and a GPCR-Autoproteolysis Inducing (GAIN) domain encompassing a GPCR-proteolytic site [1]. The C-terminus of BAI1 contains a proline-rich region and a terminal Gln-Thr-Glu-Val (QTEV) PDZ-binding motif, which associates with a number of scaffolding proteins involved in intracellular signaling [1]. BAI1's TSR are required for functions as varied as the phagocytosis of apoptotic cells by macrophages, myogenesis, and the inhibition of angiogenesis (Figure 1) [1]. Cleavage of the BAI1 N-terminus releases soluble 40 and 120 kDa peptides called vasculostatins, for their anti-angiogenic activity. Overexpression of BAI1 or vasculostatin-120 can inhibit tumor growth in several cancer xenograft models *in vivo* [1]. Whether the tumor suppressor function of BAI1 is entirely dependent on its extracellular TSRs, or also involves its emerging intracellular signaling activity is currently unclear. The human *BAI1* gene is located on chromosome 8q24 and its expression is epigenetically silenced in glioma. In addition, recent discoveries have shown that the genes encoding BAI1, BAI2 and BAI3 undergo somatic mutations in several cancers, including lung, breast, ovarian and brain, suggesting that they might be tumor suppressors [1]. To further explore whether BAI1 loss might be linked to tumor development, we recently generated a line of *Bai1*-knockout (*Bai1^−/−^*) mice. The mutant mice are viable and fertile, obtained at the expected Mendelian ratio and have no obvious anatomical defects [2]. Aging the mice did not evidence any increase of cancer incidence, suggesting that loss of *Bai1* expression is not sufficient to induce tumor formation *per se* [2]. More work is needed to define whether a cancer susceptibility role for BAI1 will only be revealed when combined with alterations in other pro-tumorigenic loci.

**Figure 1:**
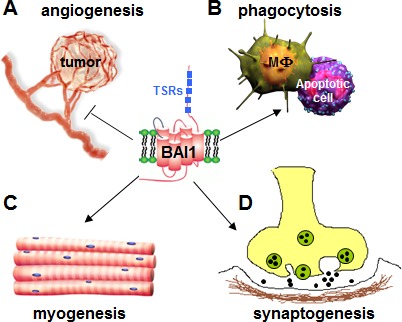
Multiple functions of BAI1 **A.** BAI1 N-terminal thrombospondin type 1 repeats (TSRs) inhibit tumor-induced angiogenesis. **B.** The TSRs on the BAI1 N-terminus interact with exposed phosphatidylserine on apoptotic cells and elicit phagocytosis by the macrophages (MΦ). **C.** BAI1 stimulates myogenesis by promoting the fusion of myoblasts into multinucleated myofibers. **D.** Deficiency in BAI1 promotes PSD-95 protein degradation at the synapses and induces enhanced long-term potentiation (LTP).

In the brain, BAI1 is enriched in neurons at the postsynaptic density (PSD), an electron-dense thickening in the postsynaptic membrane that contains over 100 synaptic proteins and regulates the strength of the excitatory signal, a phenomenon called synaptic plasticity. This suggests that BAI1 might serve as a critical regulator of synapse development, maturation and maintenance. To test this hypothesis, we used neurophysiological techniques to provide electrical stimulations to neurons in hippocampal synapses of the *Bai1^−/−^* mice and found strikingly enhanced long-term potentiation (LTP) and impaired long-term depression (LTD) [2]. LTP and LTD are the most widely studied types of synaptic plasticity and are believed to underlie multiple forms of learning and memory. Consistently, the *Bai1^−/−^* mice show severely impaired spatial learning and memory in the hidden-platform version of the Morris water maze task [2]. Synaptic plasticity can also be associated with changes in dendritic spine morphology. BAI1 interacts with a variety of proteins that exert powerful effects on dendritic spine formation. For example, BAI1-associated protein 2 (BAIAP2), also called insulin receptor substrate 53 (IRSp53), is a Rho family GTPase effector and has essential roles in filopodia formation and neuronal development [3]. Knockdown of BAIAP2 by short interfering RNAs reduces the density, length, and width of dendritic spines in neuron cultures [4]. Similarly, acute BAI1 knockdown results in a ∼50% reduction in spine density of cultured neurons [5]. Based on these results, we expected changes in the density or morphology of dendritic spines of neurons in the *Bai1^−/−^* brains. Surprisingly, we did not detect any significant changes in spine formation and/or maturation in *Bai1^−/−^* CA1 pyramidal neurons. These data suggest that morphological changes induced by BAI1 loss in dendritic spines are only observed under *in vitro* conditions, or that compensatory effects take place *in vivo* during development.

Exploring the mechanisms underlying the deficits of synaptic plasticity in the *Bai1^−/−^* mice, we found that *Bai1* deficiency promotes a rapid degradation of PSD-95, a major PSD scaffolding protein, and virally-mediated restoration of PSD-95 in the *Bai1^−/−^* hippocampus is sufficient to normalize synaptic plasticity [2]. This, along with the observation that *Bai1^−/−^* mice and the *Psd95*- mutant mice show strikingly similar phenotypes, suggest that the deficits observed in the *Bai1^−/−^* mice are due, at least in part, to loss of *Bai1*-induced PSD-95 stability. Our results have wide-ranging implications for a variety of neurodegenerative diseases. For example, Alzheimer's disease (AD) is a neurological disorder characterized by the abnormal aggregation of the amyloid beta component of the amyloid precursor protein (APP), yet APP's endogenous function remains largely unknown. Reduction in PSD-95 is among the earliest changes in synaptic composition in the APP mutant neurons [6], suggesting that dysregulation of PSD-95 may underlie AD pathogenesis. Recent studies from genome-wide transcriptome analyses indicate that BAI family mRNA expression is downregulated in large cohorts of AD patients [7]. These results, along with our observations, provide a rationale to expand the exploration of BAI1's contribution to disease pathogenesis beyond cancer, including in neurological diseases such as AD.

## References

[1] Cork SM (2011). J Mol Med.

[2] Zhu D (2015). J Clin Invest.

[3] Scita G (2008). Trends Cell Biol.

[4] Choi J (2005). J Neurosci.

[5] Duman JG (2013). J Neurosci.

[6] Almeida CG (2005). Neurobiol Dis.

[7] Webster JA (2009). Am J Hum Genet.

